# Quantitative Timed-Up-and-Go Parameters in Relation to Cognitive Parameters and Health-Related Quality of Life in Mild-to-Moderate Parkinson's Disease

**DOI:** 10.1371/journal.pone.0151997

**Published:** 2016-04-07

**Authors:** Janet M. T. Van Uem, Stefan Walgaard, Erik Ainsworth, Sandra E. Hasmann, Tanja Heger, Susanne Nussbaum, Markus A. Hobert, Encarnación M. Micó-Amigo, Rob C. Van Lummel, Daniela Berg, Walter Maetzler

**Affiliations:** 1 Hertie Institute for Clinical Brain Research, Department of Neurodegeneration, Center of Neurology, University of Tuebingen, Tuebingen, Germany; 2 DZNE, German Center for Neurodegenerative Diseases, Tuebingen, Germany; 3 McRoberts, The Hague, The Netherlands; 4 MOVE Research Institute Amsterdam, Department of Human Movement Sciences, VU University Amsterdam, Amsterdam, The Netherlands; University of Toronto, CANADA

## Abstract

**Introduction:**

The instrumented-Timed-Up-and-Go test (iTUG) provides detailed information about the following movement patterns: sit-to-walk (siwa), straight walking, turning and walk-to-sit (wasi). We were interested in the relative contributions of respective iTUG sub-phases to specific clinical deficits most relevant for daily life in Parkinson’s disease (PD). More specifically, we investigated which condition–fast speed (FS) or convenient speed (CS)–differentiates best between mild- to moderate-stage PD patients and controls, which parameters of the iTUG sub-phases are significantly different between PD patients and controls, and how the iTUG parameters associate with cognitive parameters (with particular focus on cognitive flexibility and working memory) and Health-Related-Quality of Life (HRQoL).

**Methods:**

Twenty-eight PD participants (65.1±7.1 years, H&Y stage 1–3, medication OFF state) and 20 controls (66.1±7.5 years) performed an iTUG (DynaPort^®^, McRoberts BV, The Netherlands) under CS and FS conditions. The PD Questionnaire 39 (PDQ-39) was employed to assess HRQoL. General cognitive and executive functions were assessed using the Montreal Cognitive Assessment and the Trail Making Test.

**Results:**

The total iTUG duration and sub-phases durations under FS condition differentiated PD patients slightly better from controls, compared to the CS condition. The following sub-phases were responsible for the observed longer total duration PD patients needed to perform the iTUG: siwa, turn and wasi. None of the iTUG parameters correlated relevantly with general cognitive function. Turning duration and wasi maximum flexion velocity correlated strongest with executive function. Walking back duration correlated strongest with HRQoL.

**Discussion:**

This study confirms that mild- to moderate-stage PD patients need more time to perform the iTUG than controls, and adds the following aspects to current literature: FS may be more powerful than CS to delineate subtle movement deficits in mild- to moderate-stage PD patients; correlation levels of intra-individual siwa and wasi parameters may be interesting surrogate markers for the level of automaticity of performed movements; and sub-phases and kinematic parameters of the iTUG may have the potential to reflect executive functioning and HRQoL aspects of PD patients.

## Introduction

Parkinson's disease (PD) is a chronic, progressive, multisystem neurodegenerative disease. Patients with PD show symptoms and signs in several domains (e.g. motor, cognitive and autonomic functioning), which can have an effect on Health-Related Quality of Life (HRQoL) [1]. HRQoL incorporates dimensions of physical, mental, social, and role functioning, and can include abilities, relationships, perceptions, life satisfaction and well-being [2,3].

A not yet clearly defined plethora of–primarily motor, but also non-motor–symptoms can potentially be defined with relatively simple tests, e.g. the timed-up-and-go test (TUG). The TUG measures the time required to perform a sequence of activities, including the sit-to-walk (siwa) transfer, straight walking, turning, and the walk-to-sit (wasi) transfer. These movement sequences are frequently performed in daily life. People with PD experience problems during the performance of these movements [4]. Therefore, the TUG is a suitable clinical test to assess mobility in people with PD [5–7].

Increasing evidence shows that the instrumented TUG (iTUG, TUG performed with e.g. inertial sensors) provides relevant additional information, compared to the ‘classic’ TUG that measures only total time [8,9]. These inertial sensors can register movements with e.g. tri-axial accelerometers and gyroscopes, and algorithms can then describe different sequences of this iTUG in detail. For example, a previous study showed that 17 PD patients performing the iTUG under convenient conditions had a lower range of movement during the siwa, compared to 15 age-matched healthy controls[8].

According to similar studies performed with individuals at high risk for future PD [10–12] and early clinical stages of PD [13], challenging iTUG assessment strategies may have the potential to detect deficits which are not yet visible with the clinical eye, and may therefore have a particularly high potential for treatment and prevention strategies [14]. One option is to let the participants perform the task under fast speed (FS) conditions [15].

The iTUG may also have some potential to detect cognitive disabilities associated with PD [16]. This is of relevance as (i) cognitive deficits regularly occur even at very early PD stages–in fact, there is a twofold increased risk for mild cognitive impairment for early PD patients compared to healthy controls [17]; (ii) executive function, which is most relevant for the supraspinal control of axial movements [14,18], is often particularly affected by the disease [19,20]; and (iii) the quality of learned and automatically performed movements–such as finger tapping movements, but also gait and transfer–seems to be altered in PD. In more detail, there is evidence that the execution of automatic movements (i.e. frequently performed movements, especially the movements that require less precision; during execution of automatic movements no relevant attention is directed towards the details of the movement [21]) is altered, and PD patients need ‘more conscious brain activity’ to perform automated movements [22]. Finally, as the movements investigated with the iTUG are particularly relevant for performance of daily life activities, it is tempting to hypothesize that parameters extracted out of the iTUG may be associated with HRQoL. Not surprisingly, a recent publication showed that longer time needed to complete the TUG correlates with lower HRQoL [16]. However, it is not known yet which of the TUG sub-phases are responsible for this association. This information may be relevant for optimizing therapies to keep HRQoL as high as possible in PD.

Based on the above aspects, this study focused on the analysis of the iTUG sub-phases of PD patients and controls, to

1. identify whether iTUG performed with FS is superior to iTUG performed at convenient speed (CS) to differentiate between PD patients and controls,
2. define which parameters of the iTUG sub-phases are significantly different between PD patients and controls, and
3. explore the relationships between the iTUG sub-phases and cognitive parameters (with particular focus on cognitive flexibility and working memory) as well as HRQoL.

## Methods

### Design

This prospective cross-sectional study is in accordance with principles of the latest version of the Declaration of Helsinki, and all participants signed informed consent. The board of the ethical commission of the Medical Faculty of the University of Tübingen, Germany approved the study.

### Participants

A total of 48 participants (28 PD patients and 20 controls, well matched concerning age and gender, see Table 1 for details) were recruited from the outpatient clinic of the Neurodegenerative Department of the University Hospital of Tübingen, Germany. Inclusion criteria for PD patients were diagnosis of idiopathic PD according to the UK Parkinson's Disease Society Brain Bank criteria confirmed by movement disorders specialists (DB, WM), mild-to-moderate stage as defined by a Hoehn & Yahr (H&Y) stage between 1 and 3, ability to walk and stand safely without walking aid for 30 minutes, non-invasive interventions such as Deep Brain Stimulation, a minimum score of 25 points on the Mini Mental State Examination (MMSE) [23], and no other neurological disease. Spouses of the included PD patients were asked to participate as controls; inclusion criteria here were no history of neurological diseases and also a minimum score of 25 points on the MMSE.

**Table 1:** 

	PD (n = 28)	Controls (n = 20)	P-value
Age	64.9 ±7.1	66.1 ±7.5	0.56
Gender (female) [%]	12 (43)	8 (40)	
MAS (left/right)	16/11	-	
Disease duration [years]	6.5 (±2.9)	-	
H&Y (1–5) ♦	2 (1–3)	-	
MoCA (0–30) ♦	28 (23–30)	29 (23–30)	0.61
MMSE (0–30) ♦	29 (27–30)	29 (26–30)	0.43
Delta TMT [s]	59.1 (±59.4)	47.2 (±31.8)	0.38
MDS-UPDRS III (0–132)	27.3 (±12.8)	3.8 (±4.5)	**0.00**
PIGD score (0–12)	2.4 (±1.7)	0.5 (±0.9)	**0.00**

Demographic and clinical data, presented as mean and standard deviation or percentage of total, if not otherwise stated.

^♦^Median (range). Delta TMT, Delta Trail Making Test; H&Y, Hoehn & Yahr; MAS, most affected side; MDS-UPDRS III, Movement Disorder Society- Unified Parkinson's Disease Rating Scale part III; PD, Parkinson’s disease; PIGD, Postural Instability and Gait Difficulties.

### Clinical assessment

All participants underwent an intensive clinical assessment including medical history taking, medication intake and neurological examination. Motor disability was evaluated using part III of the revised version of the Unified Parkinson's Disease Rating Scale (MDS-UPDRS)[24]. A separate Postural Instability and Gait Difficulties (PIGD) score was calculated from the items 3.10–3.12 [25]. General cognitive function was assessed with the Montreal Cognitive Assessment (MoCA) [26]. Trail Making Test (TMT) part B minus part A [27] was employed to assess cognitive flexibility and working memory [28]. The summary index (SI) as well as the mobility [29] and activities of daily living (ADL) [30] subdomains of the Parkinson's Disease Questionnaire-39 (PDQ-39), a disease-specific HRQoL questionnaire [31,32], was used to assess HRQoL. We hypothesised, based on previous research [29,30,33], that the PDQ-39 mobility and ADL domains show associations with objective mobility and balance scales. The MDS-UPDRS III and the iTUG assessments (see below) were performed during medication OFF, after withdrawal from dopaminergic medication overnight. The patients were then enabled to take their medication, before MoCA, TMT and PDQ-39 assessments were performed.

### iTUG assessment and used sensor

The iTUG, comprising a sequence of siwa, 3-meter straight walk forth, 180-degree turn, 3-meter straight walk back, and wasi, was performed twice with CS (self-selected speed), and twice with FS. The turn in one of the respective iTUG’s was performed in clockwise direction, and one with a turn in counter clockwise direction. The sequence of the four single iTUG conditions was identical for all participants.

During the assessment, all participants wore one portable, small and light-weighted inertial sensor that integrates a triaxial accelerometer and a triaxial gyroscope (DynaPort Hybrid^®^, McRoberts, The Netherlands) with a sampling frequency of 100 Hz. A detailed description of the sensor is given elsewhere[34].

### Algorithm

The algorithm used in this study has been validated in separate studies [35,36]. In brief, global turning phases were determined using the low-pass filtered and squared angular velocity around the vertical axis. Start and end temporal events of the turning phases were determined using threshold detection based on the differentiated low-pass filtered and squared angular velocity around the vertical axis. Start and end temporal events of the siwa and wasi phases were determined using peak detection of a low-pass filtered vertical acceleration signal. Maximal flexion angles of the siwa and wasi were determined using trunk angle signal. End and start temporal events of the siwa and wasi phases were determined as the first peak of the vertical acceleration signal after and before the maximum flexion angles and above the mean of the vertical acceleration signal.

### Statistical analysis

Durations of iTUG sub-phases and kinematics (angular range and angular velocity) during these sub-phases were used for analysis. Turning towards the MAS or LAS of the body did not show relevant differences between cohorts among the parameters analysed (not shown); we therefore used the mean values of these conditions for further analyses. In general, all data are presented with mean and standard deviation, or with percentage of total. If the skewness score of data was greater than 1 or -1, the median and range was used. The Shapiro-Wilk test was used to test for normality. Accordingly, Student’s t test, and Wilcoxon Rank test were used to check for significance between groups. In all analyses, significance was accepted at level p<0.05. In addition, the following statistical methods were used to answer the above-mentioned hypotheses:

The Area Under the Curve (AUC) of the Receiver Operating Characteristic (ROC) curve was used to compare discriminatory power of CS and FS.

Unpaired t-test was used to compare durations of iTUG’s sub-phases and kinematics between controls and PD patients. The paired t-test was used for comparison of lateralization aspects among PD patients.

To evaluate the automaticity of learned movements [22,37], Pearson’s correlation coefficients (r) of corresponding siwa and wasi parameters within an individual were compared between PD patients and controls.

For the association of cognitive parameters and HRQoL aspects with iTUG parameters, Pearson’s r was used. Pearson’s r can be interpreted as follows: 0: zero, 0.1–0.3: weak, 0.4–0.6: moderate, 0.7–0.9: strong, 1: perfect association among the respective parameters [38]. All parameters of the iTUG sub-phases that showed a significant correlation with cognitive parameters or HRQoL in the Pearson’s correlation analysis as well as age were included in a stepwise multivariate regression analysis.

All analyses were performed on Statistical Package for the Social Sciences (SPSS) version 22.

## Results

An overview of demographic and clinical data is presented in Table 1. The MDS-UPDRS III total score and the PIGD sub score were significantly different between PD patients and controls. No significant differences were detected for the factors age, gender, cognitive functioning (MoCA) and executive functioning (TMT A, B and delta TMT).

The AUC of the ROC curve of the FS iTUG discriminated better than CS iTUG between PD patients and controls (0.736 vs 0.699). Comparably, all durations of sub-phases of the iTUG under FS conditions discriminated better than CS condition: siwa (0.740 vs. 0.678), walk forth (0.640 vs. 0.556), turn (0.804 vs. 0.683), walk back (0.619 vs.0.596), and wasi (0.653 vs. 0.603). All further analyses were therefore performed with data obtained from the FS task.

The duration of the walking phases was not significantly different between PD patients and controls. However, the following phases yielded significant differences between cohorts:

*Siwa phase*: PD patients needed significantly more time to complete the siwa phase than controls (p<0.01). The lower mean and maximum flexion velocities attributed to this difference. In the extension movement, maximum velocity was lower in PD patients than in controls. Mean extension velocity showed a trend towards significance.

*Turning phase*: Turning duration was significantly longer in the PD cohort than in the control cohort (p<0.01). Maximum turning velocity was significantly lower in PD patients compared to controls.

*Wasi phase*: PD patients needed significantly more time to complete the wasi phase than controls (p = 0.04). In the flexion movement, PD patients showed a significantly lower range of movement than controls, as well as significantly lower mean and maximum velocities. In the extension movement, range of movement and mean velocity were significantly lower in PD patients than in controls. Maximum extension velocity was lower in PD patients (p = 0.05). Details are presented in Table 2.

**Table 2:** 

iTUG in FS condition	Controls	PD	Controls vs PD	r with Delta TMT (p-value)	r with PDQ-SI (p-value)	r with PDQ-mob (p-value)	r with PDQ-ADL (p-value)
Total iTUG duration [s]	7.8 (1.2)	9.4 (2.1)	0.00	0.49	0.58	0.71	0.59
				0.01	0.00	0.00	0.00
**Siwa** duration [s]	1.3 (0.3)	1.7 (0.6)	0.01	0.50	0.44	0.64	0.43
				0.01	0.02	0.00	0.02
**Siwa** flexion range [°]	58 (9)	54 (8)	0.09	-0.08	-0.20	-0.16	-0.29
				0.69	0.31	0.42	0.14
**Siwa** mean flexion velocity [°/s]	99.5 (23.4)	64.7 (17.5)	0.00	-0.18	-0.23	-0.17	-0.24
				0.38	0.24	0.40	0.21
**Siwa** max. flexion velocity [°/s]	120.5 (26.3)	100.0 (19.8)	0.01	0.12	-0.06	0.12	0.43
				0.56	0.75	0.55	0.02
**Siwa** extension range [°]	25 (8)	21 (10)	0.11	0.08	-0.00	-0.07	-0.02
				0.69	0.99	0.74	0.94
**Siwa** mean extension velocity [°/s]	46.3 (19.4)	35.7 (21.5)	0.08	-0.17	-0.17	-0.26	-0.18
				0.41	0.39	0.19	0.57
**Siwa** max. extension velocity [°/s]	45.7 (17.3)	33.4 (13.3)	0.02	0.19	-0.00	0.18	-0.06
				0.36	1.00	0.37	0.76
**Walk forth** duration [s]	1.2 (0.5)	1.5 (0.6)	0.16	0.33	0.51	0.51	0.42
				0.10	0.01	0.01	0.02
**Turn** duration [s]	1.9 (0.3)	2.2 (0.3)	0.00	0.48	0.39	0.54	0.45
				0.01	0.04	0.00	0.02
**Turn** max velocity [°/s]	183 (19)	151 (30)	0.00	-0.30	-0.40	-0.48	-0.35
				0.13	0.04	0.01	0.07
**Walk back** duration [s]	1.0 (0.3)	1.2 (0.4)	0.12	0.44	0.52	0.68	0.48
				0.02	0.00	0.00	0.01
**Wasi** duration [s]	1.7 (0.3)	2.0 (0.8)	0.04	0.20	0.39	0.41	0.44
				0.32	0.04	0.03	0.02
**Wasi** flexion range [°]	28 (12)	17 (9)	0.00	-0.02	-0.05	-0.12	0.00
				0.92	0.82	0.54	0.99
**Wasi** mean flexion velocity [°/s]	39.0 (22.2)	20.1 (10.5)	0.00	0.29	0.14	0.14	-0.08
				0.15	0.47	0.49	0.68
**Wasi** max. flexion velocity [°/s]	41.5 (18.5)	26.6 (10.1)	0.00	0.50	0.07	0.34	-0.03
				0.01	0.73	0.08	0.88
**Wasi** extension range [°]	48 (8)	43 (8)	0.04	-0.02	0.15	0.11	0.13
				0.90	0.44	0.57	0.50
**Wasi** mean extension velocity [°/s]	59.1 (18.1)	48.0 (15.5)	0.03	-0.03	-0.42	-0.30	-0.48
				0.90	0.03	0.13	0.01
**Wasi** max. extension velocity [°/s]	99.2 (23.9)	87.5 (17.7)	0.05	-0.20	-0.16	-0.17	-0.25
				0.32	0.41	0.39	0.21

Sensor-based measures extracted out of the instrumented Timed-Up-and-Go test (iTUG) under fast speed (FS) conditions, separately presented for Parkinson's Disease (PD) patients and controls, and their correlation with delta Trail Making Test (delta TMT, reflecting executive function), Parkinson’s Disease Questionnaire (PDQ)-Summary Index (SI, reflecting general Health Related Quality of Life, HRQoL), PDQ-mobility (reflecting the mobility domain of HRQoL), and PDQ-Activities of Daily Living (ADL, reflecting the ADL domain of HRQoL). Durations of sub-phases are presented at the right border of the first column, qualitative parameters at the left. Max., maximum; r, correlation coefficient; siwa, sit-to-walk sub-phase; wasi, walk-to-sit sub-phase.

To evaluate the level of ‘automaticity’, all corresponding siwa and wasi parameters within the individual were correlated separately for PD patients and controls. Only the parameter duration correlated more closely in the PD cohort than in controls. All other (i.e. kinematic parameters) correlated closer in the control cohort than in the PD cohort (Table 3).

**Table 3:** 

Parameter	PD	Controls
	r	r
Duration	0.37	0.23
Flexion range	**0.42**	**0.52**
Mean flexion velocity	0.19	**0.49**
Maximum flexion velocity	**0.56**	**0.64**
Extension range	0.07	0.17
Mean extension velocity	0.04	-0.21
Maximum extension velocity	0.24	**0.55**

Pearson’s correlation coefficients (r) of the corresponding parameters of the sit-to-walk and the walk-to-sit sub-phases, presented for the Parkinson’s disease (PD) and controls. Significant values are in bold. Note that PD patients had a higher correlation value than controls for the correlation of duration, but lower values for all other (kinematic) parameters.

None of the parameters explored correlated significantly with the MoCA. Conversely, siwa duration, turning duration and walking back duration, as well as the kinematic parameter wasi maximum flexion velocity were significantly correlated with the delta TMT. Details are presented in Table 2. In the stepwise multivariate analysis including all parameters of the iTUG sub-phases that were significantly associated with the delta TMT, duration of turn and wasi maximum flexion velocity remained significant. These parameters explained 38.5% of the variance of the delta TMT. Details are presented in Table 4.

**Table 4:** 

	Adjusted R^2^	Standardized beta	T	Significance
Delta TMT	0.385			
(Constant)			-2.83	**0.01**
wasi maximum flexion velocity (°/s)		0.49	2.96	**0.01**
Duration turn (s)		0.43	2.76	**0.01**
PDQ-SI	0.246			
(Constant)			-0.87	0.39
Duration walk back (s)		0.52	3.13	**0.00**
PDQ-mobility	0.545			
(Constant)			-4.17	**0.00**
Duration walk back (s)		0.48	3.21	**0.00**
Duration siwa (s)		0.39	2.63	**0.02**
PDQ-ADL	0.233			
(Constant)			-0.84	**0.40**
Duration walk back (s)		0.48	2.81	**0.01**

Stepwise multivariate regression analyses, with all iTUG parameters that showed a significant correlation with executive function or Health Related Quality of Life (HRQoL) in the correlation analyses (see also Table 2). Delta TMT, Trail Making Test (TMT-B—TMT-A); PDQ, Parkinson's Disease Questionnaire; PDQ-ADL, PDQ- Activities of Daily Living; PDQ-SI, PDQ- Summary Index.

Durations of all sub-phases except turning were significantly associated with the PDQ-SI. Out of the kinematic parameters extracted from the sub-phases, maximum turning velocity and mean extension velocity of the wasi phase showed a significant correlation with the PDQ-SI (Table 2). In the stepwise multivariate analysis duration of walking back remained significantly associated with the PDQ-SI. The parameter explained 24.6% of the variance of the PDQ-SI (Table 4).

Durations of all sub-phases correlated significantly with the PDQ-mobility subdomain. Out of the kinematic parameters, the maximum turning velocity was significantly associated with this subdomain (Table 2). In the stepwise multivariate analysis, two parameters–duration of walking back and duration of the siwa sub-phase–remained significantly associated. These parameters explained 54.5% of the variance of the PDQ-mobility subdomain (Table 4).

Durations of all sub-phases correlated significantly with the PDQ-ADL subdomain. Out of the kinematic parameters extracted from the sub-phases, the siwa maximum velocity was significantly associated with this subdomain (Table 2). In the stepwise multivariate analysis walking back duration remained significantly associated. This parameter explained 23.3% of the variance of the PDQ-ADL domain (Table 4).

## Discussion

In this study, we found that durations and kinematic parameters of sub-phases of the FS iTUG (which differentiated slightly better between PD patients and controls, than the CS iTUG) had the potential to explain different aspects of motor and executive functioning aspects of the disease, as well as HRQoL aspects of patients affected by PD.

The FS condition differentiated our mild-to-moderate stage PD patients better from controls than the CS condition. Although differences are small, we argue that the effect can be valid. In early PD, gait, transfer and balance functions are relatively well preserved and are similar to controls’ functions under convenient assessment conditions [39]. However, subtle deficits are revealed when PD patients are maximally challenged [40,41]. Moreover, the unilateral character of PD may add to the observed difference, as lateralization of motor symptoms can cause lower synchronisation between body parts [15,42].

The following observations need specific attention. In agreement with a previous study [43], PD patients showed, compared to controls, a prolonged time on the siwa phase. The significantly lower mean and maximum flexion velocity and maximum extension velocity led to this prolonged time. The best explanation is most probably the presence of hypokinesia in the PD cohort. Hypokinesia is defined as the absence or poverty of automatic movements, e.g. during sequential movements–such as siwa [44]. Also the overall changed movement pattern of PD patients could contribute to the observation. Buckley and colleagues [43] investigated siwa of PD patients and controls utilising the Vicon motion capture system. They found that PD patients rose to almost full height before taking the first step, whereas controls initiated gait closer to the point of seat-off [43]. This result suggests that PD patients have impairments in sequencing movement patterns, more specifically switching from one movement to another [44,45]. Most difficulties may be experienced during movement patterns when the movement direction is switched from flexion to extension [44], such as rising from a chair.

PD patients had longer turning duration and lower peak turning velocity than controls. This finding is in agreement with previous studies [9,46,47]. According to literature [48–50], this finding could be best explained by an impairment of the natural sequence of turning. Normally, head rotation precedes turning of the trunk and feet consecutively [48–50]. Lower interlimb synchronisation [15,42,51], balance and stability impairments [49,52], and loss of axial flexibility of the spine [53]–due to increased axial muscle tone [54]–may limit this natural sequence of turning. The increased muscle tone in PD is often most prominent in the neck [54], which may further limit the initiation of the craniocaudal turning sequence [52]. As a consequence, the environment may not be scanned as properly, and rotation not be adjusted to the surroundings as healthy people do [55].

Like the siwa phase, PD patients showed a prolonged time on the wasi phase, compared to controls. This difference was driven by significantly lower mean and maximum flexion and extension velocity. We hypothesize–as it is most probably also the case in the siwa phase–that hypokinesia explains a relevant proportion of the observation. Moreover, as the wasi phase includes a 180° turn, the lower range of motion (flexion and extension), as observed in PD patients compared to controls, may be caused by the combination of (i) the typical PD bent forward posture of the trunk, and (ii) extra caution and attention during the execution of the movements triggering extra flexion of the trunk. We hypothesize that this posture could be of advantage for PD patients, as it provides a stabilizing effect during rotating movements on the spot by leaving the displacement of the centre of mass in a relatively similar horizontal position [56], consecutively decreasing the risk of a backward fall [56,57].

Intra-individual correlations between the durations and kinematic parameters of the siwa and wasi phases suggest that PD patients perform transfers less consistent than controls. We argue that the loss of automaticity causes this loss of consistency, and that this lowered correlation values may have the potential to serve as progression markers in PD, and potentially also as outcome markers in therapy trials. Loss of automaticity is well investigated in PD patients. The posterior putamen substantially contributes to the memory of automatic movements, and the relevant dopamine depletion in this area obviously induces a failure to shift to automatic motor programs [58]. As a consequence, PD patients have to pay more attention than controls to perform automated movements [22,59]. As attention to one’s skilled performance impairs the performance [59,60] (even if this is not subjectively perceived [61]), automated movements are more inconsistent. It is tempting to speculate that higher inconsistencies of all *kinematic* parameters of the siwa and wasi, as found in PD in this study, reflect a risk factor for unsafe movements and even pre-falls and falls. The higher correlation of siwa and wasi *duration* in PD patients compared to controls does in our view not argue against this hypothesis, as it PD patients may have lower flexibility to adapt the duration of the transfers to external situations.

We could not identify a significant relation between overall cognitive function and the iTUG within our PD cohort. A previous study [62] including 197 older adults without cognitive impairment and 208 older adults with cognitive impairment (ranging from mild to severe impairment) found a relationship between overall cognitive functioning and the total TUG duration. Differences between the results of the previous study and our results may be explained by different disease populations (older adults living in home care institutions versus PD patients living at home), lower sample size in our study, by different disease entities, the relatively good cognitive state of our PD cohort, and different methods used to measure TUG (stopwatch vs sensors). Nonetheless, we found a relationship between a number of iTUG sub-phases and their kinematic parameters and working memory and cognitive flexibility measured with delta TMT. Our results are basically in line with previous studies [16,63], although these former studies did not investigate sub-phases but only total TUG duration. They independently found a relationship between total TUG duration and verbal executive function [16] and semantic fluency [63]. We found that the siwa duration, turning duration and walking back duration, and the kinematic parameter wasi maximum flexion velocity are correlated to executive function. The durations of the siwa and turning phases, and wasi maximum flexion velocity correlated strongest. The finding suggests that PD patients rely more on parameters of working memory and cognitive flexibility during the performance of complex motor tasks such as transitions and turning, than controls do [58,64]. Our hypothesis is supported by a functional magnetic resonance imaging study [22]. PD patients showed higher activity in the dorsolateral prefrontal cortex before, [65] and higher activity in the premotor cortex during execution of a sequential finger movement, compared to controls [22,66]. These changes can again be interpreted as a loss of automaticity, [58] and PD patients rely more on modulation of behaviour, allocation of simultaneous attention and regulation of response inhibition–which are all parameters of executive function–during preparation and performance of complex automated motor tasks [58,64]. In our view, our results are of particular relevance, as many of our daily behaviours are carried out automatically; therefore PD considerably affects daily functioning. If our observations are confirmed by other studies, they may eventually lead to specific (non-pharmacologic) treatment, which is e.g. already recommended in the European Physiotherapy Guideline for PD [4].

Although the duration of the walking back phase did not significantly differentiate between PD patients and controls, it correlated strongest in the multivariate analysis with HRQoL (PDQ-SI, PDQ-mobility and PDQ-ADL). It is acknowledged that gait has a pivotal role in daily functioning [67], and it has a relatively strong relationship with general HRQoL, and with the HRQoL mobility domain in particular [33]. Difficulties with ambulation is a key determinant to disability and is therefore directly related to reduced HRQoL [67]. Axial impairment frequently gives rise to gait impairments in PD patients, and it is known to constrain HRQoL [68,69].

The lack of overlap of parameters that are associated with either delta TMT or PDQ-SI argues for a weak relationship between executive functioning and HRQoL. This assumption is supported by a recent systematic review [70]. It is known that, by using cognitive therapy, mobility can improve, however the influence of these treatments on HRQoL may be weak [64]. Additionally, a recent study showed that cognitive dual task training did not improve HRQoL of PD patients during a 6-week training intervention (A. Nieuwboer, personal communication).

We acknowledge that this study has limitations. First, we focused on the investigation of mildly to moderately affected PD patients, without relevant overall cognitive impairment. Therefore, the results are not valid for the whole PD population but only for PD patients with relatively preserved cognitive function. We argue that this is a particularly interesting cohort to delineate subtle deficits; however our findings cannot be generalized to the overall PD population. Future studies should consider inclusion of severely affected and cognitively impaired patients. Second, we did not define presence of absence of mild cognitive impairment as proposed by the Movement Disorders Task force on in Parkinson's disease [71], and we did not assess the intelligence quotient, which might be associated with executive function parameters. Third, the TMT test does not cover the complete range of executive function, however in studies investigating a range of cognitive parameters, the TMT showed the strongest association with ADL functions in non-demented PD patients [72,73]. Fourth, we could not measure interlimb coordination–an interesting parameter concerning automaticity aspects–because we used only one sensor placed at the lower back. However, our aim was to use a sensor system that can easily be applied in clinical practice. Fifth, the repeated assessment of iTUGs in every study participant includes the risk of fatigue. Patients were, however, allowed to rest between trials. Sixth, patients took their PD medications during the clinical visit, after the movement assessment and before the executive function and HRQoL assessment. This approach might have influenced the relationship between these variables. Although it is to our best knowledge not yet shown that the outcome of the MoCA, TMT and PDQ-39 is significantly modified by the actual medication state in early- to mid-stage PD patients, results of this study should be interpreted accordingly. Finally, study design (cross-sectional) and sample size are considered as limitations. However, this is a hypothesis-generating study with well-matched cohorts; we therefore argue that the approach is justified.

## Conclusions

We conclude that the FS-iTUG is a particularly suitable tool for detecting subtle movement deficits in mild-to-moderate stage PD patients. The iTUG may provide information about specific aspects of hypokinesia and loss of automaticity, deficits in working memory and cognitive flexibility (even in PD patients without relevant overall cognitive impairment) and HRQoL aspects.
